# Parental participation in newborn care in the view of health care providers in Uganda: a qualitative study

**DOI:** 10.1186/s12978-024-01896-w

**Published:** 2024-10-29

**Authors:** Phillip Wanduru, Claudia Hanson, Doris Kwesiga, Angelina Kakooza-Mwesige, Helle Mölsted Alvesson, Peter Waiswa

**Affiliations:** 1 Department of Global Public Health, Karolinska Institutet, Stockholm, Sweden; 2 Department of Health Policy, Planning, and Management, School of Public Health, Makerere University, Kampala, Uganda; 3 Department of Disease Control, London School of Hygiene and Tropical Medicine, London, England; 4 Department of Pediatrics and Child Health, School of Medicine, Makerere University, Kampala, Uganda; 5 Busoga Health Forum, Jinja, Uganda

**Keywords:** Family-centered care, Newborn care, Newborn care unit, Health care providers, Qualitative research

## Abstract

**Background:**

Evidence suggests that family-centered care for sick newborns, where parents are co-caregivers in newborn care units, can result in increased breastfeeding frequency, higher weight gain, earlier discharge, and reduced parental anxiety. This study explored healthcare providers' perceptions and experiences of parental participation in care for sick newborns in the newborn care units in two high-volume maternity units in Uganda, with the aim of informing interventions that promote family-centered care for newborns.

**Methods:**

An exploratory qualitative study was conducted between August and December 2023. Sixteen in-depth interviews were held at a regional and general hospital in the rural eastern region of Uganda. The interviews were audio-recorded and then transcribed, followed by a reflexive thematic analysis approach to generate themes.

**Findings:**

We identified four key themes: (1) creating order to ensure the safety of newborns in the newborn care unit; (2) parental participation as a tool for overcoming workload in the Newborn care unit; (3) redirecting parental involvement to focus on medically endorsed newborn care practices; and (4) stress management targeting mothers to ensure newborn survival.

**Conclusion:**

Healthcare providers encourage parents to participate in caring for their newborns in the newborn care units, mainly to reduce their workload. However, our study highlights the imbalanced nature of parental involvement, where HCPs control the tasks parents can or cannot perform, essentially deploying them as "assistants" rather than equal partners, contrary to the ideals of family-centered care. Transforming the current "healthcare provider-centered" model of caring for sick newborns to one that is family-centered will require training providers on the benefits of family-centered care and developing guidelines for its structured implementation within a resource-limited setting.

## Background

A growing body of evidence suggests that family-centered or family-integrated care for sick newborns involving parents as mutual partners in planning, decision-making, and caregiving for their newborns in Newborn Care Units (NCU) has positive health outcomes [1–4]. Common elements of family-centered care include building capacity for healthcare providers (HCP), providing parents with psychological support, open communication between parents and the HCPs, and creating a favorable physical environment for parents in the NCU. Additionally, allowing parents to be continuously present and involved in planning, decision-making, and caring for their newborns is essential to family-centered care [3, 5].

The benefits of family-centered care in NCUs include increased breastfeeding frequency, faster weight gain, earlier discharge of newborns, and better parent-newborn bonding [1–4, 6–9]. Parents involved in their newborn's care experienced less stress, greater satisfaction with care, and more confidence in caring for their newborn post-discharge [6, 8, 10, 11]. However, almost all research on family-centered care for sick newborns, apart from that on Kangaroo Mother Care (KMC) specifically, comes from high-income countries.

In view of the favorable evidence, the World Health Organization (WHO) and its partners released guidelines calling for the integration of family-centered care in care for newborns in NCUs [12–14]. These guidelines are part of the broader efforts by WHO and its partners to assist countries in achieving the United Nations Sustainable Development Goals related to newborn health by promoting high-impact interventions [15]. They emphasize the importance of the parents' rights to (i) be present in the NCU, (ii) participate, and (iii) make decisions about newborn care, alongside the right to receive the psychological care they need. Many African countries have adopted these guidelines [16].

Despite the clear positive evidence and policy framework, little is known about the actual implementation experiences of family-centered care in NCUs in Low- and middle-income settings. Evidence from high-income settings indicates that the successful implementation of family-centered care is highly contextual and best defined by actors within that setting [17–19]. Operational and structural factors may influence the extent of parental participation. These include the technical capacity and attitudes of NCU staff regarding parental participation, hospital policies regarding parental participation, and the infrastructure in the NCU that supports the presence of parents and the parent's willingness to participate in care [18, 20–22].

In Uganda, there is a growing interest among the Ministry of Health and development partners in implementing family-centered care for sick newborns in NCUs [23]. A global push and other advocacy efforts for the respectful newborn care agenda complement this. However, these efforts have been limited to mentioning "family-centered care" in national Reproductive Maternal Newborn and Child Health policy documents [23, 24]. No guidelines on implementing family-centered care are available. Despite the lack of guidance, it has been a practice for many years that parents stay with their newborns in the NCU and participate in caring for them alongside HCPs. However, the experiences and scope of parental participation remain poorly understood.

As part of the efforts to inform the interventions to enhance parental involvement in line with the principles of family-centered care for sick newborns in Uganda's NCUs, in 2022, we conducted an initial study exploring the experiences and perspectives of parents regarding their participation in caring for their newborns in the NCU [25]. To complement these perspectives, this follow-on study sought to explore the perceptions and experiences of HCPs regarding parental involvement in NCUs in eastern Uganda.

## Methods

### Study design and setting

This exploratory qualitative study was conducted in NCUs of the two highest-volume public hospitals in a 14-district region of rural East Central Uganda. The hospitals include a general hospital and a regional referral hospital. Both hospitals have NCUs that are adjacent to their maternity units. The staff at the NCU in the referral hospital includes two consultant pediatricians, four neonatal nurses, two intern doctors, and two resident pediatricians. In comparison, the general hospital staff consists of a non-consultant pediatrician, a medical doctor, three neonatal nurses, and intern doctors, but it lacks resident pediatricians. The nursing staff work eight hourly shifts while the doctors work during the day and are on call at night.

The two hospitals were selected because they represent typical high-volume tertiary-level public health facilities. Health care is free at these hospitals, and they typically serve the rural and lower socioeconomic class, who cannot afford private health care. The NCUs of both facilities take care of, on average, 30 to 40 newborns at a time. Each newborn typically has a parent as a caretaker. Further details of the setting have been described elsewhere [25, 26].

### Study participants

Our study population consisted of HCPs working in the NCUs of the two hospitals. The inclusion criteria included all the HCPs in the two NCUs working full-time at the NCU for at least two months. We considered this period sufficient for gaining ample experience in dealing with parents. Sixteen HCPs, including nurses, medical doctors, and pediatricians, met this criterion.

Table 1 shows the sociodemographic characteristics of the healthcare providers participating in the study.

**Table 1:** characteristics of respondents

Respondent characteristics	Number
Age range	N = 16
20–30	5
30–40	6
50>	5
Years of working in the NCU	
2–12 months	1
12–24 months	3
24 months or more	13
Cadre	
Nurse	9
Medical officer (including residents)	5
Pediatricians	2

### Data collection

Data were collected through in-depth interviews (IDIs) using a semi-structured interview guide. The guide was created based on insights gained from our experiences working in the setting and from our previous study, upon which this study is built [25]. The guide comprised open-ended questions that asked HCPs' opinions on parents' role in caring for their newborns and the significance of their roles. We also inquired about their personal experiences relating to parents and how the organizational structure of the NCU affected how parents participated in care. Furthermore, we included questions about HCPs' experiences dealing with parents from low-income households. In our previous study, we found that many low-income families with admitted newborns faced significant challenges in interacting with HCPs [25].

In order to recruit HCPs for the interview, we reached out to the NCU in-charge, who provided us with a list of all HCPs who met the study inclusion criteria. Subsequently, PW personally invited all suitable candidates and scheduled interview dates at their convenience. To facilitate a productive conversation during the interviews with HCPs, we shared information about the study and the Interview Guide with them at least a day before the scheduled interview date [27].

The interviews were conducted in English by PW, who has a clinical background, with support from two female research assistants with a social sciences background. All interviewers have extensive experience collecting qualitative research data. The interviews lasted 40 to 60 min and were audio-recorded.

Before the interview, we comprehensively explained the study's objectives and procedures to the participants. We obtained written informed consent from participants. This study received ethical clearance from the Higher Degrees Research and Ethics Committee of Makerere University School of Public Health (SPH-2021-126) and the Uganda National Council of Science and Technology (HS2057ES).

### Research team and reflexivity

The study team included researchers with different educational backgrounds, including clinical, social sciences, and public health. Also, some were Uganda-based researchers who were familiar with and had worked within Uganda's healthcare system, and others were outsiders and unfamiliar with the system.

PW, a nurse and public health expert by training, had previously worked in the hospitals as part of a team supporting HCPs in quality improvement. This experience allowed PW to build trusted relationships with many HCPs participating in the study. This established rapport allowed participants to feel comfortable and engage in in-depth conversations. However, the preexisting relationships could have also negatively influenced the interviews in two ways. First, HCPs might have responded in ways they believed PW, known for his work in improving the quality of newborn care, would want to hear, possibly concealing what they perceive as poor clinical practices. Second, as PW was somewhat of an insider, there was a risk of assumptions being made by PW, not probing certain areas or participants withholding information because they assumed he already knew, which could limit the depth of the conversation, as described by Blythe [28]. To mitigate this, two other research assistants without prior contact with the HCPs also conducted interviews alongside PW. The data collecting team convened daily after the interviews to reflect on the emerging responses and consider whether positionalities shaped the HCPs' responses or their probing, and adjustments were made accordingly.

### Data analysis

Each data collection team member transcribed their own audio-recorded interviews. The transcriptions were uploaded into NVivo 11 Plus © (QSR International, Memphis, USA) for coding. We used reflexive thematic analysis to analyze the data following the steps described by Braun, Clarke, and others [29]. PW conducted the first steps, which included reading the transcripts thoroughly, coding the transcripts, generating categories and themes, and identifying linkages across the themes. Although PW led the coding, he continuously discussed the codes and categories with the data collectors. Following this initial process, the categories and themes were discussed with the co-authors in a series of meetings. These meetings aimed to develop themes based on understanding underlying meanings and patterns in the data, thus creating our final themes. HMA led reflective team discussions.

## Results

Four main themes were developed from analysis and are synthesized in Table 2, along with their corresponding sub-themes.

**Table 2:** Summary of themes and sub-themes

Theme 1: Creating order to ensure the safety of newborns in NCU	Spatial organization of the NCU An overcrowded and chaotic NCU creates a distracting work environment Efforts to restore order in the NCU Maintaining the ideals of high hygiene standards in the NCU
Theme 2: Parental participation as a tool for overcoming workload in the NCU	Parents adapt and learn fast when in NCU HCPs fill in the gaps whenever parents are unable to manage
Theme 3: Redirecting parental involvement to focus on medically endorsed newborn care practices	Instructive approach to providing parents with survival information Rewarding and reprimanding to ensure compliance
Theme 4: Stress management targeting mothers to ensure newborn survival	Securing breastmilk production for the survival of the newborn Providers ability to identify who needs more support

### Theme 1: Creating order in the NCU environment to ensure the safety of the newborns

HCPs aspire to have a well-organized and highly hygienic NCU environment. This theme highlights how HCPs attempted to achieve spatial organization of the NCU and maintained the ideals of high hygiene standards in the NCU.

#### Spatial organization of the NCU

The NCUs are small rooms that are often overcrowded, with 30 to 40 newborns admitted at a time. The HCPs expected each newborn to have only one parent in the NCU, but it was common for two or three additional relatives to be present, worsening the overcrowding. The congested NCU made it difficult for HCPs to physically access newborn cribs, which complicated administering treatment and routine clinical reviews. This, at times, led to some newborns missing out on care, which HCPs reported to be potentially life-threatening for some very sick newborns.

Further, the HCPs reported experiencing distress from working in the congested NCU. The overcrowded, noisy, and disorganized environment created a constant distraction, making it challenging for them to focus and provide optimal care for the newborns. The HCPs attributed some of their errors and forgetfulness to this stressful working environment.

*This place is just too crowded—overwhelmingly crowded with many babies and parents. Now, some parents are standing, and others are seated on chairs. All are having loud conversations; it is like a market. It is difficult to have some private time to focus, examine a baby, and talk to a parent at a time without being distracted by all the people and movements. Sometimes, you can miss out on important things because of all the chaos around you* (Nurse, hospital 1)

The HCPs consistently worked to reduce congestion in the NCU. To achieve this, the NCU management established guidelines restricting access to only one parent at a time. The HCPs asserted that the rule in the NCU was that each newborn should have only one parent, preferably the mother. Another parent could only replace the mother if she were too ill to be present. Besides, HCPs typically preferred the mother as the primary caregiver over any other parent because she could breastfeed the newborn.

To enforce the one-parent rule, HCPs used two main strategies: locking the door and verbally requesting extra people to leave. Although locking the door was a much simpler option, it posed a risk of locking out the mothers, who were the desired caretakers. Therefore, the HCPs used the verbal approach more frequently, even though it was strenuous for them as it required them to raise their voices often, which they found undesirable. However, they had to resort to this method to ensure the safety of the babies and prevent additional people from entering the NCU.

*I am always shouting at parents in the NCU. Getting people out of this place is almost impossible without first screaming at them. I hate to shout; I lose my voice for this. But I must shout at them otherwise; there is no order. Shouting is the only way because closing the front door may exclude mothers, such as those who leave to use the toilets. That's also not good.* (Nurse, hospital 1)

The HCPs noted that fathers (male spouses) and grandparents often ignored the restrictions on entering the NCU. They felt it was their duty to be with their spouse or daughter and the sick newborn. The HCPs reported that sometimes they would have to call on the help of the hospital security officers to persuade these relatives to leave the NCU. Despite understanding the feelings of these family members, HCPs still restricted them from accessing the NCU, a difficult but necessary part of their role. They believed that the benefits of reducing congestion in the NCU outweighed the consequences of not having a spouse or grandparent present.

*In my experience, leaving the NCU is difficult for men and grandparents to understand. They struggle to understand why they must leave the new mother and newborn baby alone when in such a vulnerable state and setting. Remember, the woman has undergone childbirth, and the baby is unwell; naturally, they do not want to leave them alone. I understand their situation. However, it is impossible to have all these people in the NCU; otherwise, it will be too congested, and we will not work. So, I say sorry, but please leave* (Nurse, Hospital 2)

#### Maintaining the ideals of high hygiene standards in the NCU

The HCPs emphasized the need for high hygiene standards in the NCU due to the extreme vulnerability of newborns to infections arising from unhygienic conditions, which could be fatal. A hospital cleaner was always on standby to ensure the NCU was clean. However, the HCPs noticed that parents entering the NCU often lacked proper hygiene. They noticed that the NCU sometimes had an unpleasant odor, indicating that some parents had not maintained proper cleanliness, which could potentially put the newborns at risk of infections. Additionally, the HCPs pointed out that some parents wore dirty clothing that was unsuitable for the NCU, especially when handling vulnerable newborns.

The HCPs were particularly concerned that many mothers in the NCU had not taken a bath since childbirth. They stated that their concern for hygiene was worse for some who had undergone surgical procedures such as episiotomies, whom they say often started to develop foul smells from not bathing regularly.

*We want to keep these babies safe from infections. These babies are at a vulnerable stage, and hygiene is essential. The problem is that you often walk into the NCU, which has an emerging foul smell, which means that caretakers are not clean. The biggest problem for me is that the mothers, after birth, do not take regular baths. Some mothers have had an episiotomy, and they have pus. They often have a terrible smell* (Nurse, hospital 2)

Efforts to ensure high hygiene standards in the NCU included regularly reminding mothers to take baths and wash their clothes frequently. The HCPs reported that, specifically for mothers, whom they sensed as unhygienic based on their smell or the dirt on their clothes, they would ask them to stop whatever they were doing and bathe.

Furthermore, they required that whenever anyone entered the NCU, they first remove their shoes and also clean their hands with alcohol. In addition, HCPs asked those wearing large clothes, such as traditional *Gomesi* (a traditional floor-length skirt with a fitted bodice), to remove them and find lighter clothing. They believed lighter clothing would result in less sweating and thus alleviate the foul smell.

### Theme 2: Parental participation as a tool for overcoming workload in the NCU

The HCPs reported that, in general, there were always more sick newborns in the NCU than they could manage effectively. They further mentioned that in addition to being so many, almost all newborns in the NCU were critically ill and needed much attention. To address this, the HCPs involved parents in caring for their newborns by assigning them some caregiving responsibilities, thereby sharing the caregiving burden.

In the NCU, parents adapted quickly to take on newborns' caregiving tasks with some support and guidance from HCPs. The parents' primary roles included:

- Feeding the newborn:
  - For stable newborns, breastfeeding directly from the mother's breast
  - For unstable newborns, expressing milk for nasogastric feeding.
- Maintaining the newborns' hygiene, including changing diapers and wiping them.
- Ensuring their safety by watching over them continuously
- KMC for low-birth-weight newborns
- Buying any medicines needed for the newborn's care.

The HCPs highlighted that the mother's primary responsibility was to feed the newborn, which is vital for survival and recovery. In the NCU, breast milk was the only accepted source of nourishment for the newborns, and HCPs strongly discouraged other feeding forms.

HCPs monitored and ensured that mothers with stable newborns were breastfeeding them. For unstable newborns unable to suckle, HCPs assist the feeding process by inserting a nasogastric tube and helping mothers feed the newborn on breast milk through the tube.

Parents were expected to maintain good hygiene for their newborns. HCPs emphasized that keeping newborns in the best possible hygienic conditions would protect them from infections. Sanitary practices included wiping the baby's skin, changing diapers as needed, and ensuring the baby was dressed in clean clothing. HCPs also helped parents with cord care, as this was a delicate task and could result in infections if not done well. Additionally, parents whose babies were preterm were expected to practice KMC for their newborns. The HCPs typically initiate KMC by showing parents how to do it, and then the parents sustain the practice themselves with HCPs watching closely.

Due to the many stories about newborns being stolen from the NCU, HCPs expected the parents to keep a watchful eye on their newborns. The HCPs reported that they made efforts to ensure that every newborn had a parent watching them to prevent theft from the NCU. They understood that this was not a task they could take on themselves as they were busy. Further, HCPs expected parents to buy medication for their newborn from external pharmacies if it was unavailable at the hospital.

HCPs noted that parents sometimes engage in activities outside the primary newborn-caring roles. For example, parents occasionally help to clean the newborn cribs and the floor. Furthermore, HCPs sometimes asked the parents to alert them when the newborn showed any signs of distress or when there was an alarm from the monitoring equipment. However, the HCPs expressed reservations about getting parents into these additional roles because they wanted the parents to focus on their primary care roles, which were already many.

"*One time, we were doing general cleaning. Then I told them you people, I am going to do my general cleaning. They said sister (nurse), we are ready to help you. They asked for gloves, and we cleaned all the incubators together. Although I accept them to help and sometimes, I ask for the help, I prefer that they focus on the newborn baby and not get carried away by these other roles"* (Nurse, Hospital 2)

### Theme 3: Redirecting parental involvement to adhere to medically endorsed newborn care practices

#### Instructive approach to providing parents with survival information

The HCPs stated that parents lacked prior knowledge and experience in caring for vulnerable newborns. They needed clear guidance to fulfill their caring responsibilities without harming the newborn. As a result, HCPs organized regular health education sessions for parents in the NCU, providing essential information to improve the chances of their newborns' survival. During these sessions, HCPs mentioned that they did most of the talking, and they demanded that parents listen attentively. They emphasized the need for parents to listen very attentively, as any mistakes they made on vulnerable newborns could potentially be life-threatening. They also stated that they used straightforward and instructive language to ensure the parents followed their guidance.

Due to the many parent-newborn pairs and a limited number of HCPs, the HCPs preferred to do the sessions in groups instead of individually. They asserted that group sessions were optimal as the basic principles (emphasized in the health education sessions) for caring for all sick newborns, including feeding, maintaining warmth, and maintaining hygiene, were the same for all newborns. Given the high turnover in the NCU, the group instructions were typically given by HCPs every morning and night shift regardless of whether they were new or existing admissions.

However, the group sessions were insufficient and needed to be supplemented with individual, one-on-one sessions focused on the newborns' specific care needs. These individual sessions were often strategically conducted while the HCPs carried out activities focused on a particular newborn, such as routine check-ups or administering medication. During these sessions, the focus was on providing parents with information about specific danger signs for their newborn and how to operate any equipment attached to the newborn. Additionally, the one-on-one health education sessions were used to address sensitive topics, such as the management of HIV-exposed newborns.

*We discuss these stigmatizing situations in the one-on-one sessions. For example, HIV-positive mothers need to be sure their babies are getting their antiretroviral medicines. It would be stigmatizing to discuss HIV in the group (Pediatrician, hospital 1)*

Although the instructions given by HCPs during group and one-on-one sessions primarily focused on telling parents what to do, we also noted a considerable focus on informing parents what not to do and dispelling common newborn care-related misconceptions. Typically, the more experienced HCPs who had a deeper understanding of the community's newborn practices and beliefs were more knowledgeable about the common misconceptions and bad practices than the younger practitioners.

Accordingly, they talked about it more than the younger HCPs. They reported that parents had diverse understandings of what they believed to be the underlying cause of their newborns' condition, with witchcraft being the most common. Such conceptions resulted in mothers seeking remedies that the HCPs perceived to be harmful to the newborn, such as the use of herbs or prematurely leaving the NCU to seek traditional care before the newborn was in a condition to be discharged. Therefore, the HCPs believed stopping parents from engaging in and believing in such harmful practices was essential. During group and one-on-one sessions, the HCPs labored to warn parents strongly against false beliefs and practices.

*We talk to them about what to do, but we also talk a lot about what is false and, thus, should not be done. They believe it is something attached to witchcraft or the baby is not being accepted in the clan; that is why they are convulsing, and that is why they are not waking up. If you do not address this, these women end up running away to witch doctors or applying herbs on the baby, which worsens the baby's outcome (Nurse, hospital 2).*

#### Rewarding and reprimanding to ensure compliance

In addition to giving clear guidance, HCPs ensured that parents complied and followed their instructions by rewarding compliance with praise and positive words. Parents who complied with all instructions earned verbal praise and recognition as "model parents" in the NCU by HCPs. They frequently asked the model parents to advise new parents and those falling short. The HCPs believed that such a role was prestigious for the parents.

On the other hand, HCPs scolded parents who did not follow their instructions. Such parents included, for example, those who did not feed the baby on time, those who did not keep the baby clean and those who refuse to do KMC. Although they recognized that scolding parents was not a good practice and could be seen as mistreatment, they felt it was necessary for the sake of safeguarding the newborns' lives.

*I must say that I understand that it is not right to shout at parents, but sometimes it is inevitable. It is for the greater good. For example, there was another woman who ultimately refused to do KMC. All she wanted was to sit and do nothing. I must quarrel with them; otherwise, the babies will not survive. Besides, many of the girls are suitable to be my daughters, so I have to tell them off until they do the right things (Doctor, Hospital 1)*

### Theme 4: Stress management targeting mothers to ensure newborn survival

The HCPs expressed an awareness that parents, especially mothers of the newborn, in the NCU experienced much stress. One of the primary concerns related to stress was how the stress would affect the mother's capacity to produce breast milk, which is primary in the survival of the newborn. Additionally, the HCPs reported that many families could not afford infant formula as an alternative to breast milk, making the mother's ability to produce enough breast milk even more critical. As a result, the HCPs took proactive steps to manage the mothers' stress by offering them words of affirmation and providing them with opportunities to express their feelings.

The HCP noted that the biggest driver of mothers' stress was a fear that the newborn might not survive. To reduce their worries, the HCP would show the new mothers the many other newborns in the NCU who were recovering despite having similar or worse conditions upon admission. The HCP encouraged the mothers to remain calm and ignore other stressors, allowing their bodies to produce breast milk, which would facilitate successful breastfeeding and, in turn, contribute to the newborn's faster recovery.

*It is a scary experience for mothers. You can see the distress on the mother's face at admission. The biggest problem with stress is that it stops the mother's breast milk production after birth. Without breastmilk, you have a complete care crisis. But here, we try to calm the mothers and show them their babies will survive. We show them other babies who came in the same situation but are improving. When they calm down, then the breast milk will come automatically *(Nurse, Hospital 1)

The HCPs highlighted groups of mothers who were especially prone to higher stress in the NCU. These included mothers who did not have family members at the hospital during this time, as well as young mothers, whom the HCPs described as confused and needing much support. With the understanding that young mothers experience more stress, HCPs informed us that they made more deliberate efforts to be friendly and give them messages of encouragement. They also noted that the young mothers were frequently unwilling to express their concerns. The HCPs were, therefore, keen to pay more attention to them.

## Discussion

This explorative study in two high-caseload NCUs in Uganda found that parents are encouraged to participate in the care of their sick newborns and have defined roles. However, they are not equal decision-makers in the planning and caring for their newborns, contrary to the ideals of family-centered care. Instead, HCPs deploy parents as "assistants" who help manage the workload of the NCU. The HCPs hold authority in the NCU. They guide parental involvement towards activities that are medically endorsed as essential for newborn survival, a strategy to improve newborn survival. Additionally, the small and overcrowded nature of the NCUs complicates the adherence to family-centered care for sick newborns.

The study finding that parents are encouraged and actively participate in caring for their sick newborns in NCU was not surprising. From our experience and past studies, we know that given the limited staff in the NCUs in Uganda, parents tend to be the de facto caregivers for their newborns [25, 30]. However, our study brings to light the imbalanced nature of parental involvement, where HCPs, who are the authority, control the extent of parental participation. This controlled parental involvement has both positive and negative impacts.

On a positive note, when HCPs guide parents to focus on implementing activities essential for newborn survival, this optimizes the delivery of safe and effective newborn care in a high-mortality yet resource-limited setting. In the typical overcrowded and understaffed NCUs in public hospitals in Uganda, HCPs often encourage parents to assist with tasks such as maintaining newborn hygiene, feeding, and monitoring to help achieve better care for newborns [30, 31]. Moreover, to ensure parental participation was safe, this study demonstrates that HCPs stayed cautious, preventing parents from engaging in harmful practices or neglecting critical lifesaving tasks. They ensured this by communicating authoritatively and enforcing compliance through appropriate reprimands or rewards. Given that harmful newborn practices, such as applying herbs and prelacteal feeding, are common in this setting [32–34], this vigilant and controlling approach by HCPs aligns with the need to safeguard the newborn's wellbeing.

However, this authoritative approach of HCPs marginalizes parents, reducing them to the role of 'assistants' and preventing them from contributing meaningfully to their newborn's care based on their values. It may also hamper their capacity to ask questions and interact freely with HCPs. While educating parents about newborn care and correcting harmful practices is part of family-centered care [3, 35], it should not be done in a way that further marginalizes already vulnerable parents. Such contradicts the fundamental principle of family-centered care: empowering parents and building their confidence to make choices as equal decision-makers in caring for their newborns [5, 36, 37].

Furthermore, in family-centered care approaches, it is typically good practice for HCPs to involve parents in tasks that align with their abilities and the support they receive from the HCPs [38, 39]. However, our study results show that HCPs appear to assign tasks with the sole aim of getting medically recommended tasks done for the newborns but without much consideration of parents' caregiving capacity at the time. Our previous study on KMC showed that some mothers were unable to hold their newborns against their chests or breastfeed due to their psychological state [10]. Therefore, we view the haphazard assigning of tasks to parents as a detrimental practice.

We noted various examples of HCPs being rude toward parents, such as shouting at parents to get out of the NCU or to make them follow other instructions. The HCPs consider shouting at parents acceptable if it ultimately leads to compliance with procedures that benefit the newborns' wellbeing. Normalizing mistreatment in the name of saving lives has been identified in Africa, particularly in the context of childbirth care [40–42]. This study highlights that mistreatment also extends to the NCU.

Additionally, the NCUs are small and have limited space to accommodate the high number of parents and their newborns, which poses a significant challenge for integrating families into NCUs. The study's findings show that HCPs were enforcing restrictions that limit parents who access the NCU, primarily because of the limited space available. Uganda's public hospital NCUs have consistently struggled with the issue of limited space, which is also associated with the high burden of sick newborns [25, 30, 31, 42]. It remains a complex problem because public hospitals rarely have additional space for expansion, and constructing new spacious NCUs is expensive.

A frequently claimed reason for restricting access to the NCU was the fear that overcrowding would lead to transmitting infections to vulnerable newborns. The evidence regarding the possibility of parents bringing infections to the NCU remains controversial. Some studies found that restricting parents helped avert infections [43, 44], but other studies do not support that restrictions have a role in infection prevention [45–47]. However, all studies on this topic are done in high-income settings. Studies to determine the infection risk when allowing more than one family member in the NCU in LMIC are urgently needed to fill this gap.

The restriction of access to the NCU to only mothers may be a setback to male involvement efforts in reproductive health in Uganda. Male involvement in reproductive health remains low, and it has been an area of prioritization by the government, donors, and programs [48, 49]. The advantages of having fathers in the NCU include better father-baby bonding, less anxiety for both parents, and better emotional and physical support for the mother, amongst other factors [50]. We, therefore, see these restrictions as key missed opportunities for male engagement.

### Implications for policy and practice

Our study findings have clear implications for policy and practice that could facilitate the shift from the rhetoric of family-centered care for sick newborns toward its actual implementation in Uganda. Key strategies include training HCPs to raise awareness of the benefits of family-centered care, developing implementation and training guidelines, and reducing overcrowding in NCUs to foster safe parental involvement.

Since implementing family-centered care for sick newborns is a relatively new model of care in the NCUs in Uganda, raising awareness about its benefits among HCPs is crucial. Drawing from our experience in Uganda, particularly from introducing KMC practice, we have learned that building a critical mass of HCPs who understand the benefits of an intervention is essential for creating champions, generating demand, and, eventually, willingness to implement the intervention. We therefore recommend organizing physical or online training workshops to educate the HCPs on the principles, content, and advantages of family-centered care for sick newborns. Studies have shown that when HCPs are trained in family-centered care and understand the benefits, they are more willing to adopt the practice [51–54].

Another key recommendation is for the Uganda Ministry of Health to collaborate with stakeholders in developing guidelines for implementing and training in family-centered care in NCUs. These guidelines should outline a feasible structured approach for HCPs to establish positive collaborative partnerships with parents in the NCU. For example, they should emphasize respectful communication, actions that empower parents to make decisions about their newborns, and addressing the physical and psychological needs of parents in the NICU, among other best practices in family-centered care for sick newborns. We strongly recommend adopting a co-creation approach involving close collaboration with HCPs, health managers, experts from successful family-centered care packages, and parents with NCU experience in developing these guidelines to enhance the feasibility and appropriateness of the guidance.

Furthermore, the issue of overcrowding in NCUs is a significant barrier to safe parental involvement in newborn care. The NCUs are often small and congested, making it difficult to involve parents in caring for newborns. The extent to which overcrowding may be a factor in the spread of infection also needs to be investigated within the context of allowing fathers' participation.

As an immediate solution, we recommend that hospital and health system managers prioritize finding local solutions to reduce congestion in the NCUs, as they have been created to support KMC [31]. From our experience, what we have seen work includes expanding the current NCUs to create more space. However, we recommend that each hospital does what is appropriate and feasible within their setting to reduce congestion in the NCUs.

## Methodological considerations

This study had several strengths. The research was designed based on previous studies and was thus grounded in the immediate realities of the NCU. We followed a non-traditional practice of sharing the questions with the HCPs who were to be interviewed a day before the interview, and this added strength and thoughtfulness to their responses. Further, we conducted a reflective analysis with a diverse team of local Ugandan and international researchers, including experts in medicine and social sciences. This multidisciplinary team allowed for enriched interpretations by incorporating a variety of perspectives. Additionally, the study's findings are likely transferable to other low-resource hospitals serving poor rural populations, which we consider a key strength.

However, there was a potential limitation. The initial coding and theme development were done by PW, who was closely connected to the setting and had a clinical background. This connection could have introduced bias, interpreting the findings primarily from a clinical and insider's perspective. To mitigate this, emerging ideas from the data were regularly reviewed and discussed with the entire research team, leading to the final themes. The process was also closely monitored by two experienced qualitative researchers—HMA, a medical anthropologist based in Sweden, and DK, a social scientist from Uganda—to ensure that critical perspectives were maintained.

## Conclusions

The study suggests that HCPs encourage parental participation in the NCU and depend on parents to help manage the NCU workload. However, HCPs control what parents can or cannot do in the NCU. This "controlled parental participation" ensures that parents strictly engage in activities that are implementing activities essential for newborn survival. While this "controlled participation" can optimize care in resource-limited settings, it reduces parents to mere "assistants" rather than co-decision makers, which contradicts the goals of family-centered care. Efforts to shift from the current HCP-centered approach to one that is family-centered should start with educating HCPs to make them more aware of the benefits of family-centered care. The Uganda Ministry of Health and its partners should also develop guidelines supporting the structured implementation of family-centered practices in NCUs.

## Data Availability

No datasets were generated or analysed during the current study.
